# Perception of Individual and Joint Action in Infants and Adults

**DOI:** 10.1371/journal.pone.0107450

**Published:** 2014-09-09

**Authors:** Anne Keitel, Wolfgang Prinz, Moritz M. Daum

**Affiliations:** 1 Centre for Cognitive Neuroimaging, Institute of Neuroscience and Psychology, University of Glasgow, Glasgow, United Kingdom; 2 Research Group ‘Infant Cognition and Action’, Max Planck Institute for Human Cognitive and Brain Sciences, Leipzig, Germany; 3 Department of Psychology, Max Planck Institute for Human Cognitive and Brain Sciences, Leipzig, Germany; 4 Developmental Psychology, Department of Psychology, University of Zurich, Zurich, Switzerland; Birkbeck, University of London, United Kingdom

## Abstract

Infants and adults frequently observe actions performed jointly by more than one person. Research in action perception, however, has focused largely on actions performed by an individual person. Here, we explore how 9- and 12-month-old infants and adults perceive a block-stacking action performed by either one agent (individual condition) or two agents (joint condition). We used eye tracking to measure the latency of participants’ gaze shifts towards action goals. Adults anticipated goals in both conditions significantly faster than infants, and their gaze latencies did not differ between conditions. By contrast, infants showed faster anticipation of goals in the individual condition than in the joint condition. This difference was more pronounced in 9-month-olds. Further analyses of fixations examined the role of visual attention in action perception. These findings are cautiously interpreted in terms of low-level processing in infants and higher-level processing in adults. More precisely, our results suggest that adults are able to infer the overarching joint goal of two agents, whereas infants are not yet able to do so and might rely primarily on visual cues to infer the respective sub-goals. In conclusion, our findings indicate that the perception of joint action in infants develops differentially from that of individual action.

## Introduction

Practically from birth, infants observe the behaviour of the people around them, and they learn to anticipate the goals of others’ actions during their first year of life (e.g., [1]). Recently, interest in how infants passively perceive others’ *interactions* emerged, that is, actions performed jointly by more than one person (e.g., [2]). It is as yet an unsolved question whether the perception of joint action is essentially consistent with individual action, or whether they follow different developmental trajectories. For example, if a 12-month-old infant is able to understand an action performed by one agent, does he or she understand the exact same action if it is performed by two agents? The present research aimed to investigate this question by presenting infants and adults with a block-stacking action that was either performed by one or two agents.

An important aspect of one’s own performance, as well as action perception, is the anticipation of the future end state of the action [3]. The occurrence of anticipatory gaze shifts indicates that an observer has built a representation of the observed action goal that allows one to predict the outcome of the action before it is completed, and it is typically modulated by infants’ production skills with the respective action (e.g., [4]). The anticipation of actions has been investigated extensively both in adults [5]–[9] and infants [1], [4], [10]–[12]. In these studies, the perception of individually performed manual actions was assessed such as reaching-to-grasp an object [4], [11], moving an object into a container [1], or eating [13]. Depending on the task, infants start to anticipate action goals at around 6 months [14], [15], and by the end of their first year of life, infants are able to anticipate the goal of many manual actions (e.g., [1], [4]). However, in our social world, actions are often performed jointly by more than one person. These joint actions vary from involving two interaction partners (e.g., in a face-to-face conversation) to a multitude of cooperating or competing interaction partners (e.g., in musical or sport performances). Although frequently observed in everyday life, little research has addressed the question of how infants and adults passively perceive these interactions.

### 1.1. Joint action in adults and infants

Adults generally coordinate their actions easily to achieve a joint goal such as preparing a dinner together (for an overview see [16]). To do so, adults represent and predict not only their own actions, but also their interaction partner’s actions [16], [17]. Performance of simple tasks is often improved if another person is present, a phenomenon called social facilitation (e.g., [18]), whereas having more than one person involved in more complex tasks can lead to performance impairment [19]. Studies on task sharing have also demonstrated more specific interferences in situations where two adults acted according to complementary task rules (e.g., [20], [21]). In general, adults are exceptionally capable of actively engaging in coordinated joint action.

Infants participate in parent-child exchanges practically from birth (for an extensive overview of the first two years see [22]). During the first months of life, these face-to-face interactions become increasingly coordinated with respect to their timing and structure [23]. Importantly, in early interactions, infants are not required to represent the interaction partner’s intentions or goals [22]. In the second half of the first year of life, the adult-infant dyads include external objects and events, which is referred to as joint attention [24]. Around their first birthday, infants also begin to initiate joint action [24], and between 14 and 18 months children begin to autonomously engage in coordinated joint action with adults [25]–[27]. Thus, during the first year of life, infants participate in joint action, but it is only by the second year of life that they actively coordinate their actions with others.

### 1.2. Perception of nonverbal and verbal interactions

Infants do not only engage in joint action with their parents or their siblings. Given their limited motor repertoire in the first year of life, they also observe interactions between other people without being directly involved. For example, most infants have ample opportunity to observe their parents having a conversation, or helping each other in the kitchen. It remains a largely unexplored question how infants in their first year of life perceive jointly performed actions, at an age when they are not yet able to engage in coordinated joint action themselves.

In one of the few studies that investigated the perception of a nonverbal interaction, 6- and 12-month-olds were presented with videos of one agent feeding another [28]. The 12-month-olds anticipated the goal of the feeding action (i.e., that food would be brought to the mouth of the second agent), whereas the 6-month-olds did not. By contrast, 6-month-old infants anticipated that food would be brought to the mouth if one agent fed herself [13]. These studies suggest that 6-month-olds are able to anticipate an individually performed feeding action, but not yet an interactively performed one. It is important to note, however, that these results have to be compared carefully due to different visual and timing aspects of the stimuli (e.g., position of goals, pace of movements, etc.), which occur naturally in unrelated studies. A further aspect that has been investigated is the role of infants’ experience when observing manual interactions. Comparable to infants’ anticipation of individual actions, their perception of interactions seemed to depend on their own active experience with the manual action [2]. Regarding experience with joint action, it has been demonstrated that 10-month-olds were able to infer the joint goal of two collaborative partners if they actively experienced the joint action prior to observing it in a habituation paradigm [29]. Without this active experience, the joint goal could only be inferred by 14-month-olds [30]. It has also been shown that 14-month-old infants formed expectations about communicative gestures and subsequently performed interactions [31]. Furthermore, 18-month-olds inferred a joint goal that two agents performed sequentially [32]. It is also noteworthy that, in the related field of verbal interactions (i.e., conversations between two agents), it has been demonstrated that infants anticipated the course of a conversation at least to some extent [33], [34]. Although the above described studies investigated the perception of interaction, they do not answer the question of whether the perception of joint action is essentially different from that of individual action in infants and adults. In order to investigate just this, we conducted a study in which we systematically manipulated the number of agents involved.

### 1.3. The present study

In the present study, we presented infants and adults with an action that can easily be performed by one or two agents and that is familiar to infants: building a tower of wooden blocks, or “block-stacking”. We tested 9- and 12-month-old infants, when practically no coordinated joint action capabilities are present (see [22]), and adults who are typically very skilled at coordinating their actions with others (e.g., [16]). These age groups were chosen to contrast participants with very little and very much experience in joint action in a first attempt to systematically answer the research question. The participants observed videos of a toy tower being built by either one agent (individual condition) or alternately by two agents taking turns (joint condition). We analysed the arrival of participants’ gaze shifts at goals (gaze latency). If infants were able to anticipate an action performed jointly as soon as they are able to anticipate the same action performed individually, there should be no difference in gaze latency between conditions. If, however, the perception of individual and joint action developed differentially, for example, depending on their own experience, infants should show earlier gaze latency in the individual condition. We did not expect gaze latency differences between conditions in the adult group, because adults are exceptionally capable of coordinating their actions with others.

### 1.4. Joint action and visual attention

A secondary aim of the present study was to analyse gaze characteristics that indicate overt visual attention. Individual and joint actions naturally differ with respect to the visual complexity of the observed scene; with an increasing number of agents the complexity of the visual scene increases as well. To investigate the effect of visual complexity, we used two measures to explore the participants’ attention during the perception of the actions. It has been shown that fixation duration decreases with visual complexity, whereas the number of eye movements increase [35]–[37]. Thus, shorter fixation durations and more eye movements in the joint condition than the individual condition would indicate an effect of visual complexity on eye movements. This, in turn, could affect participants’ gaze latency towards action goals. Apart from these general measures of visual attention, we analysed how much time participants spent looking at the agent(s) or the goal areas to further support the interpretation of gaze latency results.

## Method

### 2.1. Participants

The final sample consisted of 23 9-month-old infants (*M* = 9 months 6 days; range: 9; 2 to 9; 12; 12 female), 23 12-month-old infants (*M* = 12 months 2 days; range: 11; 15 to 12; 15; 11 female), and 14 adults (*M* = 23.4 years; range 21 to 28; 6 female). Seven more 9-month-olds and seven more 12-month-olds were tested but did not complete enough trials to be included in the analyses due to fussiness in one or both conditions. One additional adult participant had to be excluded from analyses due to a technical error. All infants were born at full term. Infants received a toy for their participation, and adults received monetary compensation.

### 2.2. Ethics statement

The study was approved by the local ethics committee at the University of Leipzig, and conducted in accordance with the Declaration of Helsinki. Written informed consent was obtained from the adult participants and from infants’ parents.

### 2.3. Apparatus and stimuli

**Figure 1:**
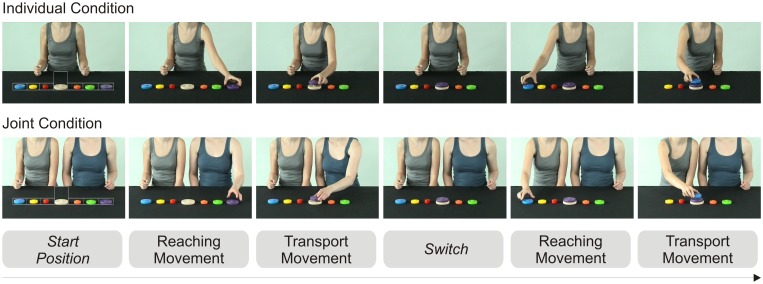
Snapshots of individual and joint conditions. The white boxes in the left panel illustrate AOIs for each goal area. The average duration (and standard deviation) in the individual condition were *M* = 970 ms (*SD* = 66 ms) for reaching movements, and *M* = 987 ms (*SD* = 62 ms) for transport movements. In the joint condition these were *M* = 990 ms (*SD* = 39 ms) for reaching and *M* = 987 ms (*SD* = 142 ms) for transport movements.

Two videos were recorded, showing how a tower of coloured wooden blocks was stacked and unstacked by either one agent (individual condition) or two agents (joint condition; see Figure 1). In both conditions, the complete tower consisted of six blocks, which were initially placed to the left and right of the base. The agent(s) alternately reached for (and grasped) one block at a time from the left and from the right, and placed it on the base (“stacking”). Once the tower was complete, the blocks were replaced in their initial position in reverse order (“unstacking”). The presented action involved one overarching goal (to build a tower) and a number of sub-goals (to reach for a block; to stack it). For the analyses, a sub-goal was defined as the area that each movement (either a reaching or a transport movement) was aimed at. Participants’ gaze behaviour towards a total of 24 reaching and transport movement sequences (i.e., sub-goals or trials) per video was analysed. To increase the participants’ attention towards the stimulus presentation, a “swooshing sound” was presented during the transport sequences. During the recording session, a metronome ticked at the rate of 1 Hz to pace the actors’ movements, and to make the timing in the two conditions as similar as possible. Accordingly, the tower was built rhythmically, and each movement (reaching for a block; transporting a block) lasted approximately 1 s (see Figure 1 for details). The difference in the mean durations of movements between the two conditions was minimal (10 ms, i.e., 0.5%). The length of each action sequence video was approximately 40 s. Conditions only differed in the number of agents; all other aspects (number and position of blocks, timing of movements, background, lighting, etc.) were analogous.

Videos were presented on a 17-inch monitor and subtended a visual angle of approximately 28.3°×19.8°. Gaze was measured using a remote corneal reflection eye tracker (Tobii 1750, Stockholm, Sweden; sampling rate: 50 Hz; software: ClearView 2.7.1) with an infant add-on (precision: 1°, accuracy: 0.5°). We used a 9-point-infant calibration.

### 2.4. Procedure

Written informed consent was obtained from the adult participants and from infants’ parents prior to testing. After the calibration sequence, which took approximately 30 s, videos of the two conditions were presented. Order of conditions was counterbalanced across participants. Before the start of each video, a salient attention grabber was shown (videos of colourful toys that moved and made sounds). After watching the action sequence videos in both conditions, the presentation of each video was repeated in order to collect more valid trials. This resulted in a possible number of 48 trials per condition (96 in total), depending on the participants’ attention. The stimulus presentation took approximately 3 min.

### 2.5. Data analysis

Raw data files can be found in Data S1. Gaze data was analysed using Matlab 7.1 (The MathWorks). Areas of Interest (AOIs) surrounded the positions of the blocks as well as the tower (see white boxes in Figure 1). AOIs for the block positions ranged from 4.8° to 5.1° horizontal visual angle and covered a vertical visual angle of 2.2°. The tower AOI covered a visual angle of 4.7°×4.9°.

We computed the arrival of gaze shifts at goal AOIs relative to the arrival of the moving hand for each trial. Positive values represented anticipatory gaze shifts whereas negative values represented reactive gaze shifts. A gaze shift was classed as anticipatory if the gaze reached the correct goal AOI before the hand did. The time interval for anticipatory gaze shifts began with the movement of the hand and ended with the arrival of the hand at the goal area. At this point, the time interval for reactive gaze shifts began; it ended 1 s after the movement was finished. An individual trial was considered to be valid if a gaze shift was preceded by a fixation at the previous AOI (i.e., the starting point of the hand movement) for at least 100 ms [34]. This ensured that actions were observed attentively. Only participants with at least 12 valid trials (6 per condition) were included in final analyses. On average, 9-month-olds provided 40.6 (*SD* = 13.4), 12-month-olds 50.3 (*SD* = 21.2), and adult participants 70.6 (*SD* = 22.2) valid trials.

General measures that quantify visual attention are mean fixation duration and “number of eye movements” [35]–[37]. First, we calculated mean fixation durations using fixation data provided by the data acquisition software (ClearView 2.7.1). Shorter fixation durations have been shown to indicate an effect of increased visual stimulus complexity on eye movements [35], [37]. Second, the number of eye movements was operationalized as number of fixations because fixations and saccades usually alternate (cf. [38]). Similarly to the measure of fixation duration, more fixations, and therefore more eye movements, have been found to indicate an effect of visual complexity [36], [37]. Because there were differences in the duration participants watched the videos, we calculated the number of fixations per second, including only the time that participants looked at the screen.

We further analysed how much time participants spent looking at the goal areas (t_goal_) in relation to the time they spent looking at the body areas (t_body_). This “goal focus” was calculated as t_goal_–t_body_/t_goal_+t_body_ (cf. [39], [40]). This resulted in an index of normalised differences between −1 and 1, where positive values indicated that participants looked longer at the goal area, whereas negative values indicated they looked longer at the body area. These normalised and normally distributed values could then be used to perform an Analysis of Variance (ANOVA). In order to make both conditions comparable, the size of the body areas was identical.

## Results

### 3.1. Gaze latency

**Figure 2:**
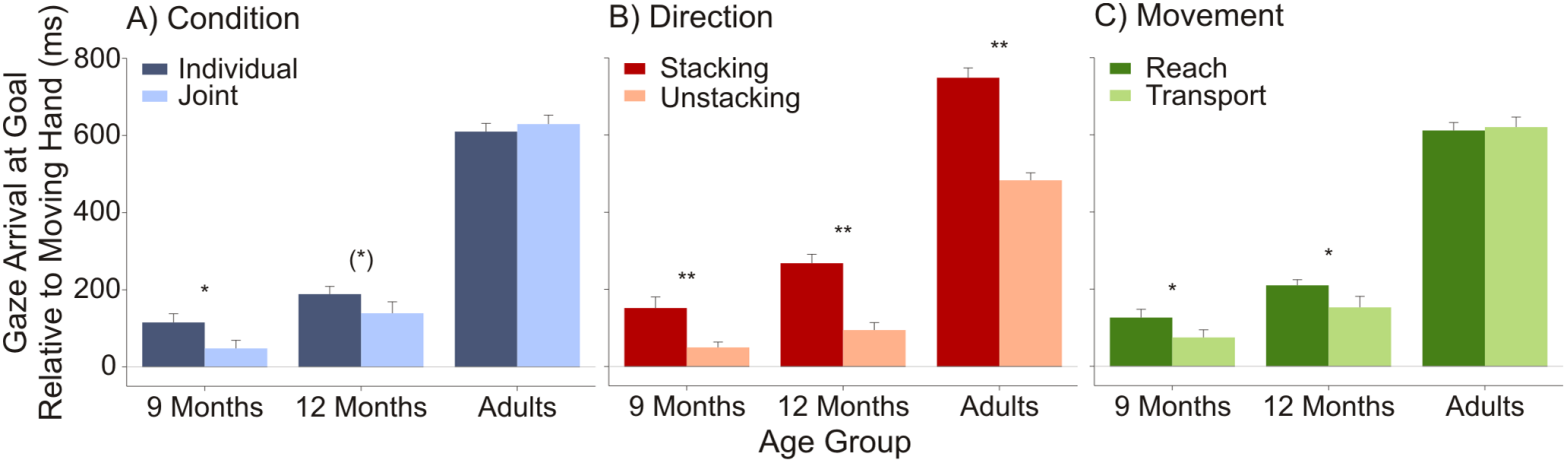
Mean gaze latency towards goals for all age groups. Mean gaze latencies are illustrated (A) in both experimental conditions, (B) for stacking direction, and (C) for movement type (with standard errors). Grey line at zero displays arrival of the hand at goal areas. Positive values indicated that gaze was anticipatory. Asterisks denote difference between a) individual and joint conditions, b) the two different directions, and c) both movement types (**: p<.01; *: *p*<.05; (*): *p*<.10).

Initial analyses did not suggest any evidence for a main effect or interaction effects of video presentation order (all *p*s>.32); those data were thus collapsed. Infants’ and adults’ gaze behaviour was anticipatory on average in both conditions (see Fig. 2 and Table 1). Performed *t*-tests against zero confirmed that participants of all age groups shifted their gaze to the action goals significantly ahead of the agent’s hand, both, in the individual condition (9-month-olds: *t*(22) = 5.13, *p*<.001, *d* = 1.07; 12-month-olds: *t*(22) = 9.45, *p*<.001, *d* = 1.97; adults: *t*(13) = 28.54, *p*<.001, *d* = 7.63) and in the joint condition (9-month-olds: *t*(22) = 2.28, *p* = .03, *d* = 0.48; 12-month-olds: *t*(22) = 4.73, *p*<.001, *d* = 0.99; adults: *t*(13) = 27.14, *p*<.001, *d* = 7.25).

**Table 1:** Mean values and standard deviations of gaze latency (in ms) in both conditions for infants and adults.

	Individual		Joint	
	*M*	*SD*	*M*	*SD*
9 Months	115.47	107.85	48.12	101.25
12 Months	188.88	95.84	139.40	141.45
Adults	609.99	79.96	629.44	86.78

Positive values indicated that gaze shifts were anticipatory on average.

A 3×2 (Age [9 months, 12 months, adults]) × Condition [individual, joint]) ANOVA with gaze latency yielded significant main effects of age, *F*(2,57) = 167.89, *p*<.001, η^2^_G_ = .80, and condition, *F*(1,57) = 4.50, *p* = .04, η^2^_G_ = .004, as well as a marginally significant interaction between both, *F*(2,57) = 2.59, *p* = .08, η^2^_G_ = .005 [generalised eta squared values are presented to ensure comparability with other studies, see 41, 42]. The main effect of age was caused by significant differences between all age groups (all *p*s<.009, Bonferroni-corrected); participants anticipated action goals faster the older they were. Paired *t*-tests showed a significant difference between the individual and the joint action condition in 9-month-olds, *t*(22) = 2.40, *p* = .03, *d* = 0.50, a marginally significant difference in 12-month-olds, *t*(22) = 2.07, *p* = .05, *d* = 0.43, and no difference in adults, *p*>.34. Thus, infants showed faster gaze latencies in the condition with one agent, whereas adults anticipated both conditions equally fast. This pattern was confirmed non-parametrically: Eighteen 9-month-olds showed faster anticipations in the individual condition, compared with only 5 who did so in the joint condition, χ^2^(1) = 7.35, *p*<.01. In the group of 12-month-olds, 15 out of 23 children anticipated actions faster in the individual condition, χ^2^(1) = 2.13, *p* = .14, as did 6 out of 14 adults, *p* = .59.

We further explored how the different types of stacking direction (stacking vs. unstacking) and movement (reach vs. transport) affected gaze latency. Stacking the blocks was anticipated faster than unstacking by all age groups (all *p*s<.003, Figure 2b); and infants, but not adults, anticipated reaching faster than transport actions (infants: *p*s<.05; adults: *p* = .67, Figure 2c). Further analyses, for example, of condition and stacking direction or movement type, were not recommended because not all participants delivered data in the corresponding trials, and often only a single trial was acquired; these limitations would lead to highly unreliable results.

### 3.2. Analyses of overt visual attention

**Figure 3:**
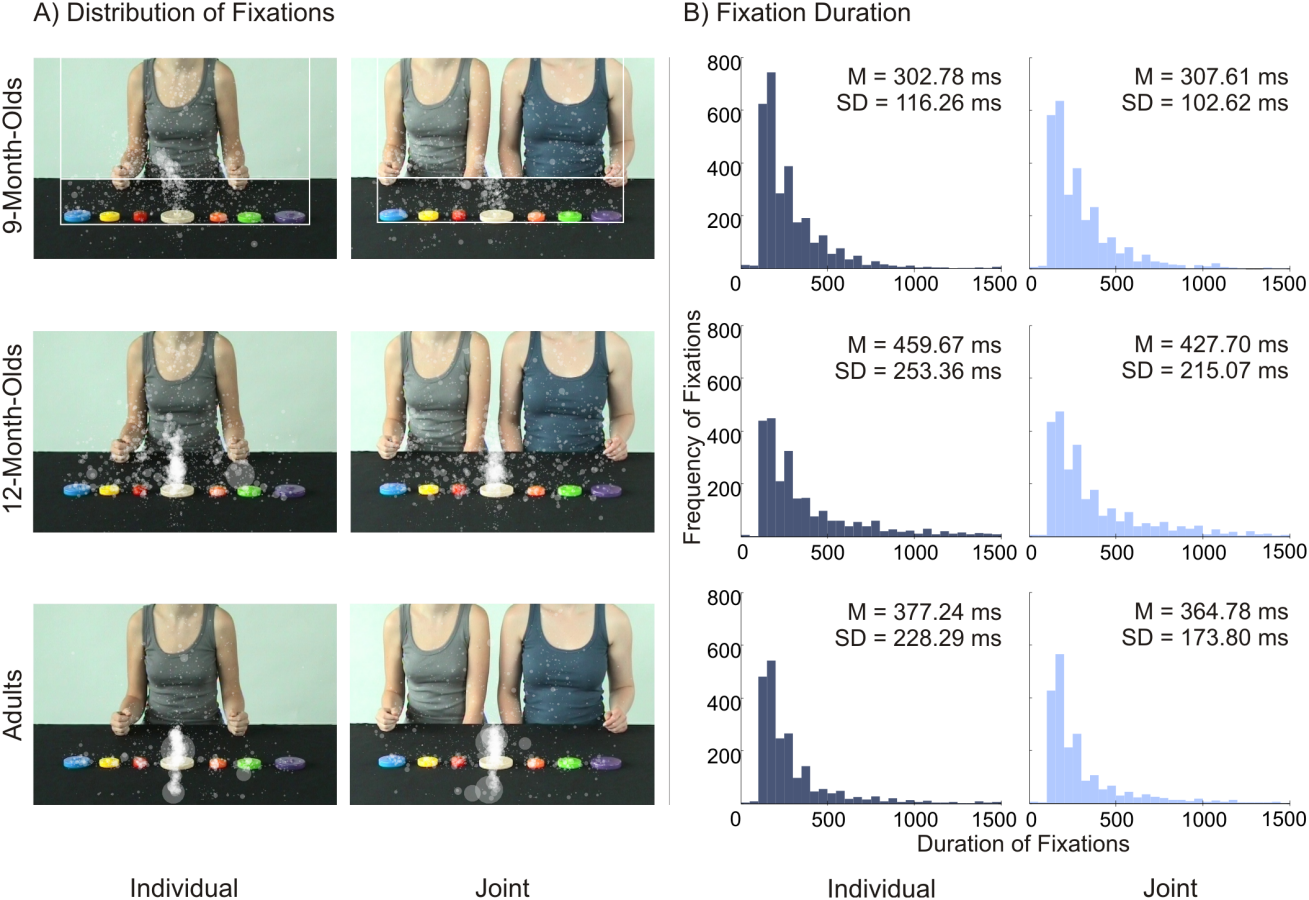
Spatial and temporal distribution of fixations. (A) Screenshots of action sequence videos with spatial distribution of participants’ gaze fixations in both conditions for 9-month-olds (top row), 12-month-olds (middle row), and adults (bottom row). Each transparent dot displays a fixation; its size indicates the fixation duration. The white boxes in the first row illustrate AOIs for goal areas and body areas. (B) Histogram of fixation duration in both conditions for 9-month-olds (top row), 12-month-olds (middle row), and adults (bottom row). Bin size is 50 ms. Mean fixation duration and standard deviations are indicated.

Figure 3B displays histograms of fixation duration in the individual and joint condition for all age groups (along with the spatial distribution of fixations illustrated in Figure 3A). A 3×2 (Age [9 months, 12 months, adults]) × Condition [individual, joint]) ANOVA with mean fixation duration yielded a significant main effect of age, *F*(2,57) = 3.29, *p*<.05, η^2^_G_ = .099, and no further effects (all *p*s>.24). Bonferroni-corrected post-hoc *t*-tests between age groups showed that 12-month-olds had longer mean fixation durations than 9-month-olds, *p* = .04, and no significant differences between infants and adults (both *p*>.74). Furthermore, a 3×2 (Age×Condition) ANOVA with fixations per second (see Table 2) yielded no significant main effects or interactions (both effects with condition: *p*s>.39; age effect: *p*>.11).

**Table 2:** Mean values and standard deviations of fixations per second and goal focus values in both conditions for infants and adults.

	Fixations per second				Goal focus			
	Individual		Joint		Individual		Joint	
	*M*	*SD*	*M*	*SD*	*M*	*SD*	*M*	*SD*
9 Months	2.19	0.67	2.18	0.61	0.19	0.24	0.11	0.24
12 Months	1.80	0.61	1.82	0.52	0.42	0.27	0.33	0.27
Adults	2.16	0.69	2.07	0.64	0.65	0.25	0.55	0.32

Positive goal focus values indicated that participants looked longer at the goal area than the body area.

**Figure 4:**
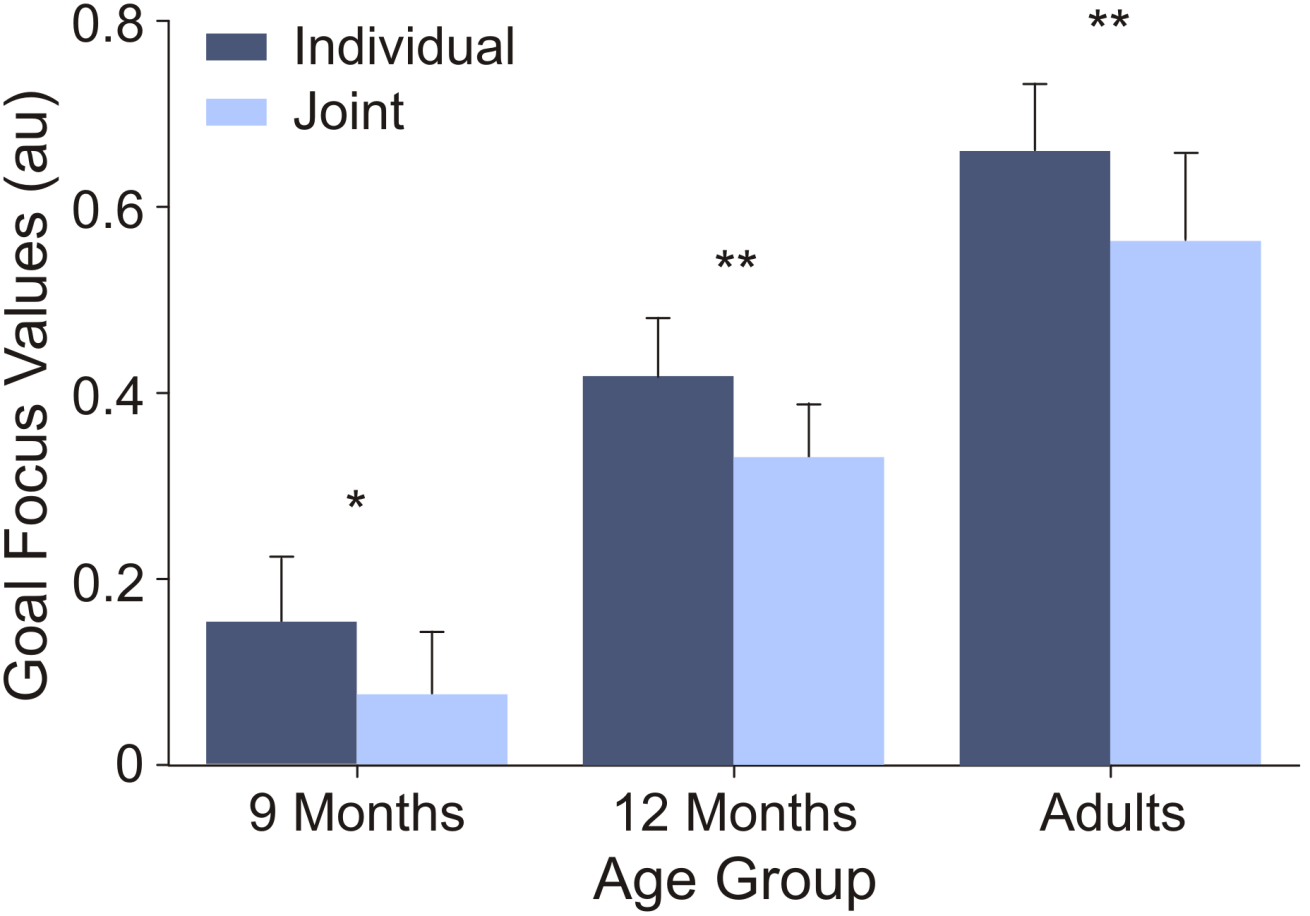
Goal focus. Normalised difference between time gazed at goal areas and time gazed at body areas. Positive values indicated that participants looked longer at goal areas than body areas (**: p<.01; *: *p*<.05).

The goal focus values for participants of all age groups were positive, indicating that they looked longer at goal areas than body areas (see Figure 4). A 3×2 (Age×Condition) ANOVA with goal focus yielded a main effect of age, *F*(2,57) = 14.27, *p*<.001, η^2^_G_ = .317, a main effect of condition, *F*(2,57) = 21.06, *p*<.001, η^2^_G_ = .001, and no significant interaction (*F*<1). Bonferroni-corrected post-hoc *t*-tests showed that the older the participants the longer they looked at goal areas, with significant differences between all age groups (all *p*s<.04). Furthermore, participants of all age groups looked longer at the body area in the joint than in the individual condition (all *p*s<.04).

## Discussion

The aim of the current study was to explore how the perception of individual and joint actions develops. Accordingly, we presented infants and adults with the same block-stacking action that was performed by either one or two agents. The main findings were that 1) adults anticipated both conditions equally fast, and they generally initiated gaze shifts towards action goals very quickly, and 2) infants anticipated action goals in the individual condition faster than the joint condition, and their gaze shifts towards goals were initiated later than those of adults. Furthermore, general measures of visual attention indicated no differences between conditions. However, participants of all age groups spent more time looking at the agents in the joint condition than the individual condition. One approach that can possibly explain the present findings is that adults and infants represented the observed actions on different hierarchical levels, namely the levels of overarching goals or sub-goals [43]. On a higher level, the overarching goal of our agent(s) was to alternately build a tower from the left and right, and this was identical in both conditions. However, if the actions were represented on the lower level of sub-goals, some differences would arise between conditions. The sub-goals were performed by either one agent or two different agents. The latter case resulted in less certainty about which agent would act. Furthermore, there was an inevitable increase in visual stimulus complexity in the joint condition, which could possibly affect participants gaze behaviour, particularly if no overarching goal representation was present. Thus, depending on whether the observed action was processed on the basis of the overarching goal or on the level of sub-goals, the conditions were either comparable or quite different.

### 4.1. Adults are able to represent joint goals

The adults in our study did not show differential gaze behaviour towards the action goals in the individual and joint condition. This suggests that they inferred the overarching goal of the agent(s) to build a tower of blocks. This higher-level representation could then be used to quickly anticipate sub-goals in a top-down manner in both conditions. It has been shown that adults usually make use of higher-level information, such as goals and intentions, that guide their anticipatory gaze shifts [44]. Such a higher-level representation leads to a fast initiation of gaze shifts because the location of the next sub-goal can be inferred before the agent has started a movement. It is thus partly independent of low-level visual information such as movement kinematics or visual stimulus complexity. Remarkably, adults showed no difference in gaze latency between conditions although their goal focus indicates that they spent more time looking at the body area (i.e., the agents) in the joint condition than in the individual condition. This can be interpreted in favour of top-down processing: Because adults knew in advance when and where to shift their gaze, they could spend more time exploring the two agents in the joint condition but were still able to anticipate the action goals equally well as in the individual condition.

There is, however, an alternative explanation as to why adults did not show differential gaze behaviour in the individual and joint condition: Adults could have performed at ceiling because the observed action was undoubtedly quite simple. This could have covered up underlying differences between conditions. It cannot be ruled out that adults would show delayed initiation of gaze shifts if observing a more demanding joint action. This remains subject to further research. However, adults are generally able to represent overarching, joint goals [16], so that a comparable gaze behaviour towards individual and joint action seems likely even in a more demanding task.

### 4.2. Infants are able to represent individual sub-goals

The infants in our study anticipated individual action faster than joint action. This suggests that the perception of joint action develops differentially from that of individual action. One interpretation to explain this finding is that infants could not benefit from a representation of the overarching joint goal in the same way as adults. Such an interpretation is supported by studies showing that infants in their first year of life are usually not yet able to infer [29] or anticipate joint action [2]. Without such a representation, gaze could not be guided towards sub-goals in a top-down manner. Instead, infants probably had to infer the sub-goal of each reaching or transport movement in a bottom-up manner while the actions were in progress, based on observable information. Indeed, infants in their first year of life have been found to represent the sub-goals of an action, instead of the overarching goal [45]. Furthermore, if children aged 9 and 12 months learned the goal of an animated agent, they subsequently anticipated the agent to choose a goal based on its previous movement path, whereas children aged 3 years, and adults, made predictions based on the agent’s previous goal [10]. Thus, infants seem to rely primarily on low-level visual cues that need to be analysed instantaneously, such as a path, or a trajectory [46]–[49], or the hand aperture in reaching actions [12], [50]. This would lead to later initiation of gaze shifts in the joint condition for a number of reasons. First, if no overarching goal representation was present, infants could not know which agent would act, and this uncertainty would delay the initiation of gaze shifts. Second, related to the first point, the corresponding representation of the agent and the agent’s goal could only be “activated” after she had started moving, because the observer had to wait for the necessary information to unfold. And third, such a switching between the representations of the two agents would lead to a processing delay that could affect gaze latency (e.g., [51]). Infants (and adults) spent more time looking at the agents in the joint condition than in the individual condition. For adults, this did not have consequences for gaze latency because their top-down processing, using the overarching goal, facilitated the anticipation of the next sub-goal. For infants, however, who relied more on the bottom-up analysis of agents’ behaviour, this would be likely to contribute to prolonged processing times to detect where to look next. Taken together, the present data suggest that infants’ gaze shifts were guided predominantly bottom-up by low-level visual information that allowed them to infer the agent(s) sub-goals. This led to a generally later initiation of gaze shifts and a differential perception of individual and joint action.

An alternative interpretation of the infants’ results is that slower gaze latencies in the joint condition are solely a consequence of increased visual distraction or longer processing times due to increased visual complexity. We do not intend to exclude this possibility altogether, but this interpretation seems unlikely for three reasons: First, general measures of visual attention (fixation duration and number of eye movements) did not indicate differences between conditions. These measures have been shown to be sensitive to visual stimulus complexity [35]–[37]. The fact that participants showed neither shorter fixation durations nor more eye movements in the joint condition suggests that the two agents in the joint condition did not elicit visual distraction per se, and visual complexity as such did not influence their eye movements. Second, the infants, as well as the adults, looked longer at two agents in the joint condition than at one agent in the individual condition, but this resulted only in later gaze shifts in the joint condition in the infant groups. This pattern suggests differential processing in infants and adults, which can be accounted for by low-level (bottom-up) processing in infants and higher-level (top-down) processing in adults. And third, previous studies have shown that infants with no coordinated joint action experience were indeed unable to infer the joint goal of two agents (cf. [2], [29]), which is in line with our interpretation that infants’ gaze patterns indicated a lack of inference concerning joint goals. Nonetheless, to resolve the issue further it could be beneficial to test an additional condition in future studies where two people sit next to each other but only one of them performs the action.

### 4.3. From low-level to higher-level processing

In the present study, the infant groups anticipated goals in the individual condition faster than in the joint condition, and this difference was more distinct in the younger infant group. This indicates differential developmental trajectories for the perception of individual and joint action. As described previously, infants probably could not make use of a representation of the overarching joint goal of two agents, whereas adults could. These findings suggest that the younger the infants, the more they depended on observable visual information (e.g., movement kinematics) to infer an action goal. This low-level visual information is less important in top-down processing where the goal is inferred before a movement has started. One of the key reasons for the development from predominantly low-level to higher-level processing is very likely experience with manual actions on the one hand, and joint action on the other hand. Such a link between anticipatory gaze shifts and experience has been shown in infants [2], [4], [15] and adults (e.g., [52]). It is to be expected that during their second year of life, children learn to anticipate joint action as well as individual action because they become more experienced in autonomously coordinating their actions with others [22]. Indeed, this notion is corroborated by findings showing that 14- and 18-month-olds could infer a joint goal [30]–[32]. The 12-month-olds in our study already showed earlier gaze latencies and a less distinct difference between conditions than the 9-month-olds. This suggests that the gaze latency measure reflects a gradual progress (as opposed to an all-or-nothing principle) from having no experience to being experienced. Due to their extensive active experience, adults were able to infer overarching joint goals and were less dependent on low-level visual information. It is has been shown, however, that adults still made use of low-level information, when a priori predictions were not possible, for example when they observed unusual or unpredictable actions [8].

Furthermore, an important factor that contributed to our results is the general development of eye movement control. Different types of eye movements, such as saccade latency or smooth pursuit, improve continually during infancy [53], [54] as well as childhood [55], [56], which is probably due to cortical maturation [54], [55]. Such a general improvement of eye movement control very likely contributed to faster gaze latencies with age. However, it cannot account for the differences between the individual and joint condition in infants.

### 4.4. Influence of salience and experience on goal anticipation

In another line of results, we found differences between the two directions of stacking (stacking vs. unstacking), and the two movement types (reach vs. transport). Stacking was anticipated faster by all age groups than unstacking. During stacking, all sub-goals were defined by salient goals (i.e., the coloured blocks during reaching, and the tower during transport actions). During unstacking, the blocks were replaced in their initial location but there was no visible goal for these transport actions, which led to later initiation of gaze shifts [57]. This result emphasises the impact of salience on goal anticipation [11].

Furthermore, infants but not adults anticipated reaching faster than transport actions. This was probably due to a lack of active experience in infants, and the impact of experience on anticipatory gaze (e.g., [4]). The ability to reach emerges at 3 or 4 months of age [58], which means that the 9- and 12-month-old infants in our study had had some experience with reaching actions. The ability to stack blocks, however, develops at around 12 months (e.g., [59]), which means that our infants had had little to no experience. This difference in active experience between the movement types most likely led to a differential perception of reaching and transport actions. It is noteworthy that this experience with *individual* action also seemed to affect the perception of *joint* action, which suggests an interplay of different experience types during action perception (see [2]). Adults had already gained extensive experience in reaching and all sorts of manipulative behaviour, including block-stacking, so they perceived these actions similarly.

An interesting detail of our results is that even the 9-month-olds anticipated action goals on average. Usually, this gaze behaviour is rarely found in infants below 12 months of age (but see [14], [15]). In our study, the rhythmic turn-taking nature of movements could have supported infants’ anticipatory gaze shifts [60], because it could have given an indication of which side of the screen was more likely to be relevant, thus narrowing location options to those within that half of the screen.

It is further important to note the bystander nature of the paradigm used in the present research. Participants observed the actions passively without being involved. The obvious benefit of this approach is that we were able to investigate infants that were not yet capable of engaging in joint action themselves. At the same time, infants might have been more attentive and motivated to make sense of our block-stacking if they had been involved.

## Conclusions

The perception of joint action in development is likely to be influenced by a complex interplay between experience with individual action and joint action on the one hand, and characteristics of the stimuli, such as visual complexity and salience, on the other hand. This leads to the finding that infants in their first year of life anticipate individual and joint action differentially. Infants might not yet be able to infer the overarching joint goal of two agents and have to make use of low-level visual information. Adults, by contrast, anticipate individual and joint goals equally fast, possibly because they are able to infer the overarching joint goal of two agents. This development from low-level to higher-level processing is most likely due to first-hand experience in coordinated joint action.

## Supporting Information

Data S1 **Raw files of eye tracking data of all participants.**
(ZIP)
